# An investigation of home advantage in the Summer Paralympic Games

**DOI:** 10.1007/s11332-017-0393-2

**Published:** 2017-08-30

**Authors:** Darryl Wilson, Girish Ramchandani

**Affiliations:** 1 Sheffield Hallam University, Academy of Sport and Physical Activity, A129 Collegiate Hall, Collegiate Crescent, Sheffield, S10 2BP UK; 2 Sport Industry Research Centre, Sheffield Hallam University, A118 Collegiate Hall, Collegiate Crescent, Sheffield, S10 2BP UK

**Keywords:** Home advantage, Disability sport, Performance, Paralympics, Multi-sport events

## Abstract

**Purpose:**

There is a paucity of home advantage research set in the context of para-sport events. It is this gap in the knowledge that this paper addresses by investigating the prevalence and size of home advantage in the Summer Paralympic Games.

**Methods:**

Using a standardised measure of success, we compared the performances of nations when competing at home with their own performances away from home in the competition between 1960 and 2016. Both country-level and individual sport-level analyses were conducted for this time frame. A Wilcoxon signed rank test was used to determine whether there was a genuine difference in nations’ performance under host and non-host conditions. Spearman’s rank-order correlation was run to assess the relationship between nation quality and home advantage.

**Results:**

Strong evidence of a home advantage effect in the Summer Paralympic Games was found at country level (*p* < 0.01). When examining individual sports, only athletics, table tennis, and wheelchair fencing returned a significant home advantage effect (*p* < 0.05). Possible explanations for these findings are discussed. The size of the home advantage effect was not significantly correlated with the quality or strength of the host nation (*p* > 0.10).

**Conclusion:**

While our results confirm that home advantage is prevalent in the Summer Paralympic Games at an overall country level and within specific sports, they do not explain fully why such an effect does exist. Future studies should investigate the causes of home advantage in the competition and also draw comparisons with the Summer Olympic Games to explore any differences between para-sport events and able-bodied events.

## Introduction

There is a paucity of research on home advantage in para-sport events targeted at elite athletes with a disability. To date, there has been a solitary study that has attempted to investigate its prevalence in a para-sport competition. Wilson and Ramchandani [1] analysed home advantage in the Winter Paralympic Games and recommended that future research should apply similar methods to the Summer Paralympic Games, to improve our understanding of this phenomenon in an under-researched sporting context. Our paper is the first attempt to directly measure the size of the home advantage in the Summer Paralympic Games. Our research had three specific aims: (1) to compare the overall performance of nations in the Summer Paralympic Games when competing at home and away from home; (2) to examine sport-specific variations in home advantage in the competition; (3) to explore the relationship between host nation strength and the magnitude of any home advantage effect. In the rest of the introduction, we review pertinent academic literature on home advantage in Olympic and Paralympic sports and then proceed to provide an overview of the Summer Paralympic Games.

The prevalence of home advantage is well documented in professional team sports that are played on a balanced home and away schedule [2]. On the other hand, the evidence for home advantage in both individual sport and unbalanced competition is less clear. A review by Jones [3] concluded that home advantage is not a major factor in individual sports, with the exception of sports that are subjectively evaluated. There is now a critical mass of published academic literature concerning home advantage in international multi-sport events. However, these studies are seldom cited, or analysed as a separate category, in literature reviews [3–6]. Most of the home advantage studies that are set in the context of multi-sport events tend to focus on the Olympic Games. One of the first formal attempts to investigate the prevalence of home advantage in a multi-sport event context was a study by Clarke [7]. His macro-level analysis revealed that 14 of the 17 countries to have hosted the Summer Olympic Games between 1896 and 1996 had won their greatest ever percentage of available medals at home. He also found that host countries typically won a greater percentage of medals at home compared with both their historical away average as well as their average in the Games immediately before and after their home games.

The prevalence of home advantage in the Olympic Games has subsequently been verified by other researchers. Balmer, Nevill, and Williams [8] observed a significant overall home advantage in the Winter Olympic Games during the period 1908–1998 when all events were combined while controlling for nation strength, changes in the number of medals on offer, and the performance of non-host nations. Thereafter, the same authors carried out a study to assess the significance of home advantage for five event groups selected from the Summer Olympic Games between 1896 and 1996 [9]. They found highly significant home advantage in event groups that were either subjectively judged (boxing and gymnastics) or relied on subjective decisions (team games), whereas little or no home advantage was observed for the two objectively judged groups (athletics and weightlifting). Yet another study by the same authors examined the number of medals won by the 14 countries that had hosted the Summer Olympic Games since the second world war until 2008 [10]. Using a logit regression model, they estimated the host nation’s odds of winning medals will increase in proportion (ratio) to 1:2.05 relative to its historical away average. Pettigrew and Reiche [11] used a linear fixed effects model to analyse home advantage in the Summer Olympics during 1952–2012 and in the Winter Olympics during 1952–2014. Pooling together the Summer and Winter results, they found that Olympic hosts tend to increase their number of gold medals by 4.4 and their total medals by 7.4 relative to their medal count in the previous Olympics 4 years earlier, albeit they acknowledge that these results are not statistically significant (*p* > 0.10) as “a consequence of a small sample size and a lack of statistical power” (p. 8). Recently, Franchini and Takito [12] provided evidence for the home advantage effect in five combat sports—boxing, fencing, judo, taekwondo, and wrestling—contested during the Olympic Games between 1996 and 2012 for total number of medals, gold and silver medals, but not for bronze medals. Akin to the assertion of Balmer et al.’s study [9], they contended that the home crowd support and its effects on referees’ decision was the main explanation for the home advantage effect in these sports. Wilson and Ramchandani [1] were the first to examine home advantage in the Paralympic Games. They found clear evidence of a home advantage effect in the Winter Paralympic Games at country level and in the sports of alpine skiing and cross country skiing. However, whether or not home advantage exists in summer para-sports is still unknown. It is this gap in the scientific knowledge that this paper attempts to address by focussing on the Summer Paralympic Games.

There have been 15 editions of the Summer Paralympic Games between 1960 and 2016 and 14 different nations have hosted the competition, as shown in Table 1, with Great Britain and USA co-hosting in 1984. Great Britain and USA are also the only nations to have hosted two editions of the competition in the time frame examined. The data in Table 1 also illustrate that there has been considerable growth in participation and in the structure of the competition, since the inaugural Summer Paralympic Games held in Rome in 1960. The 2016 edition in Rio de Janeiro featured 4328 athletes representing 160 nations contesting 528 events across 22 sports.

**Table 1:** History of the Summer Paralympics

Year	Host nation	Host city	Sports	Events	Nations	Participants
1960	Italy	Rome	8	113	17	209
1964	Japan	Tokyo	9	143	20	266
1968	Israel	Tel Aviv	10	188	28	775
1972	West Germany	Heidelberg	10	188	42	922
1976	Canada	Toronto	13	448	41	1271
1980	Netherlands	Arnhem	13	590	42	1651
1984	Great Britain (1)	Stoke Mandeville	18	975	54	2105
	USA (1)	New York				
1988	South Korea	Seoul	18	733	60	3042
1992	Spain	Barcelona	16	489	83	2999
1996	USA (2)	Atlanta	19	519	104	3255
2000	Australia	Sydney	19	550	123	3879
2004	Greece	Athens	19	519	135	3808
2008	China	Beijing	20	472	146	4011
2012	Great Britain (2)	London	20	503	164	4245
2016	Brazil	Rio de Janeiro	22	528	160	4328

Six sports have been contested in every edition of the competition: archery, athletics, swimming, table tennis, wheelchair basketball, and wheelchair fencing. The number of events contested in these sports is presented in Table 2. Overall, 5140 of the 6958 events contested between 1960 and 2016 (74%) have been in two sports, namely, athletics (41%) and swimming (33%).

**Table 2:** Events contested by sport in the Summer Paralympics

Year	Athletics	Swimming	Table tennis	Wheelchair fencing	Archery	Wheelchair basketball	Other sports	Total
1960	25	62	11	3	8	2	2	113
1964	42	62	12	7	12	2	6	143
1968	70	68	15	10	13	2	10	188
1972	73	56	19	11	12	2	15	188
1976	208	146	28	14	18	2	32	448
1980	275	192	32	17	15	2	57	590
1984	449	345	44	15	18	2	102	975
1988	345	257	37	14	9	2	69	733
1992	214	163	30	14	7	2	59	489
1996	210	168	28	15	8	2	88	519
2000	234	169	30	15	7	2	93	550
2004	194	166	28	15	7	2	107	519
2008	160	140	24	10	9	2	127	472
2012	170	148	29	12	9	2	133	503
2016	177	152	29	14	9	2	145	528
Overall	2846	2294	396	186	161	30	1045	6958

## Methods

The results of each edition of the Summer Paralympic Games between 1960 and 2016 were sourced from the historical results archive of the International Paralympic Committee (https://www.paralympic.org/results/historical) and recorded in SPSS (version 24). Our approach to define nations’ performance and calculate home advantage in this study was compliant with that used by Wilson and Ramchandani [1] in their analysis of the Winter Paralympic Games; hence, direct comparisons of our findings can be made. As illustrated by the data presented in Table 2 previously, there has been considerable fluctuation in the total number of events contested in the Summer Paralympic Games over time, ranging from a high of 975 in 1984 to a low of 113 in 1960. The number of events contested within the different sports has also not been the same throughout. Therefore, using absolute measures of performance such as the gold medal count or the total medal count does not control for the number of medals on offer or for the performance of non-hosting nations. For these reasons, we measured performance by: first, converting the number and type of medals won by each nation in a given edition into points (gold = 3, silver = 2, and bronze = 1) and second, expressing those points as a proportion of the total number of points won by all competing nations in that edition. This performance measure is henceforth referred to as ‘market share’. For example, in the 2000 Summer Paralympic Games, the host nation Australia won 63 gold medals (equivalent to 189 points), 39 silver medals (78 points), and 47 bronze medals (47 points). The total number of points won by Australia at home in 2000 was, therefore, 314 (i.e., 189 + 78 + 47). The total number of points awarded in that edition taking into account the number of events contested and medals awarded to all nations was 3306. This means that Australia’s overall home edition market share in 2000 was 9.50% (i.e., 314/3306).

To obtain a measure of home advantage, we compared each nation’s home market share with its own average market share in the editions immediately before hosting and immediately after hosting. For example, Australia’s market share in 1996 (pre-home) and 2004 (post-home) was 7.27% and 6.15%, respectively, an average of 6.71%. Therefore, its performance at home in 2000 was 2.79 percentage points better than its average pre/post-home performance (i.e., 9.50%–6.71%). Computing home advantage scores in this way ensured that less successful host countries were not unfairly compared with more successful host countries and avoided biased estimates of home advantage. Consistent with previous research on multi-sport events [1, 7–16], countries that did not host the Summer Paralympic Games were excluded from the analysis, because they had no home performances to compare with their away performances. In instances where there was no valid pre-home or post-home data (i.e., pre 1960 for Italy and post 2016 for Brazil), only the available away (pre or post) data point was utilised for comparison with their respective home performances in the home advantage calculation. The 1964 hosts, Japan, did not compete in 1960, and therefore, there was no valid pre-home (away) data point for comparison in this case. Because Great Britain and USA both hosted the 1984 Summer Paralympics, the pre-home and post-home comparator editions are the same, 1980 and 1988, respectively.

For the sport-specific analysis, archery, athletics, swimming, table tennis, wheelchair basketball, and wheelchair fencing were included, because these were the six sports that have been contested in every edition of the Summer Paralympics and they also account for the vast majority of events contested in the competition since 1960 (see Table 2). Preliminary analysis of the data showed that the home advantage residuals for host nations overall and for each sport were not normally distributed, as detected by a Shapiro–Wilk test (*p* < 0.05). For this reason, and taking into account the small sample size (*n* = 16), a Wilcoxon signed rank test was used to determine whether there was a genuine difference in nations’ performance under host and non-host conditions. Spearman’s rank-order correlation was run to assess the relationship between team quality and home advantage.

## Results

Table 3 compares the home market share of each host nation to its own away market share in the editions immediately before (pre-home) and after (post-home) hosting the competition. As mentioned previously, there is no away comparator for Italy pre 1960, Japan pre 1964, and Brazil post 2016. On 13 of the 16 occasions, nations performed better when competing at home, as indicated by the positive residuals in the final column of Table 3. This represents an overall home advantage prevalence rate of 81.25%. The only exceptions were the Netherlands in 1980, USA in 1996, and Great Britain in 2012. For these nations, their home market shares were inferior to the average of their pre-home and post-home market shares. The median difference between home and away performance was 2.11 percentage points. A Wilcoxon signed rank test confirmed that the median of differences between nations’ home and away performances was significantly greater than zero (*Z* = 2.792, *p* = 0.005).

**Table 3:** Home and away market shares of host nations in the Summer Paralympics

Year	Host nation	Pre-home (%)	Home (*H*) (%)	Post-home (%)	Avg. pre/post-home (*A*) (%)	Difference (*H* − *A*) (%)
1960	Italy	N/A	27.17	10.41	10.41	16.75
1964	Japan	N/A	2.01	1.58	1.58	0.43
1968	Israel	4.50	10.44	4.92	4.71	5.73
1972	West Germany	6.23	12.30	8.38	7.31	5.00
1976	Canada	3.08	6.26	8.82	5.95	0.31
1980	Netherlands	8.14	5.93	5.22	6.68	−0.75
1984	Great Britain (1)	6.81	11.55	8.62	7.71	3.84
	USA (1)	12.38	14.10	12.30	12.34	1.76
1988	Korea	0.11	4.75	2.73	1.42	3.34
1992	Spain	2.09	6.94	6.88	4.49	2.45
1996	USA (2)	12.69	9.44	6.65	9.67	−0.23
2000	Australia	7.27	9.50	6.15	6.71	2.79
2004	Greece	0.70	1.25	1.51	1.10	0.15
2008	China	10.02	16.12	16.23	13.13	2.99
2012	Great Britain (2)	7.55	7.62	9.86	8.71	−1.08
2016	Brazil	3.27	4.05	N/A	3.27	0.79

**Fig. 1:**
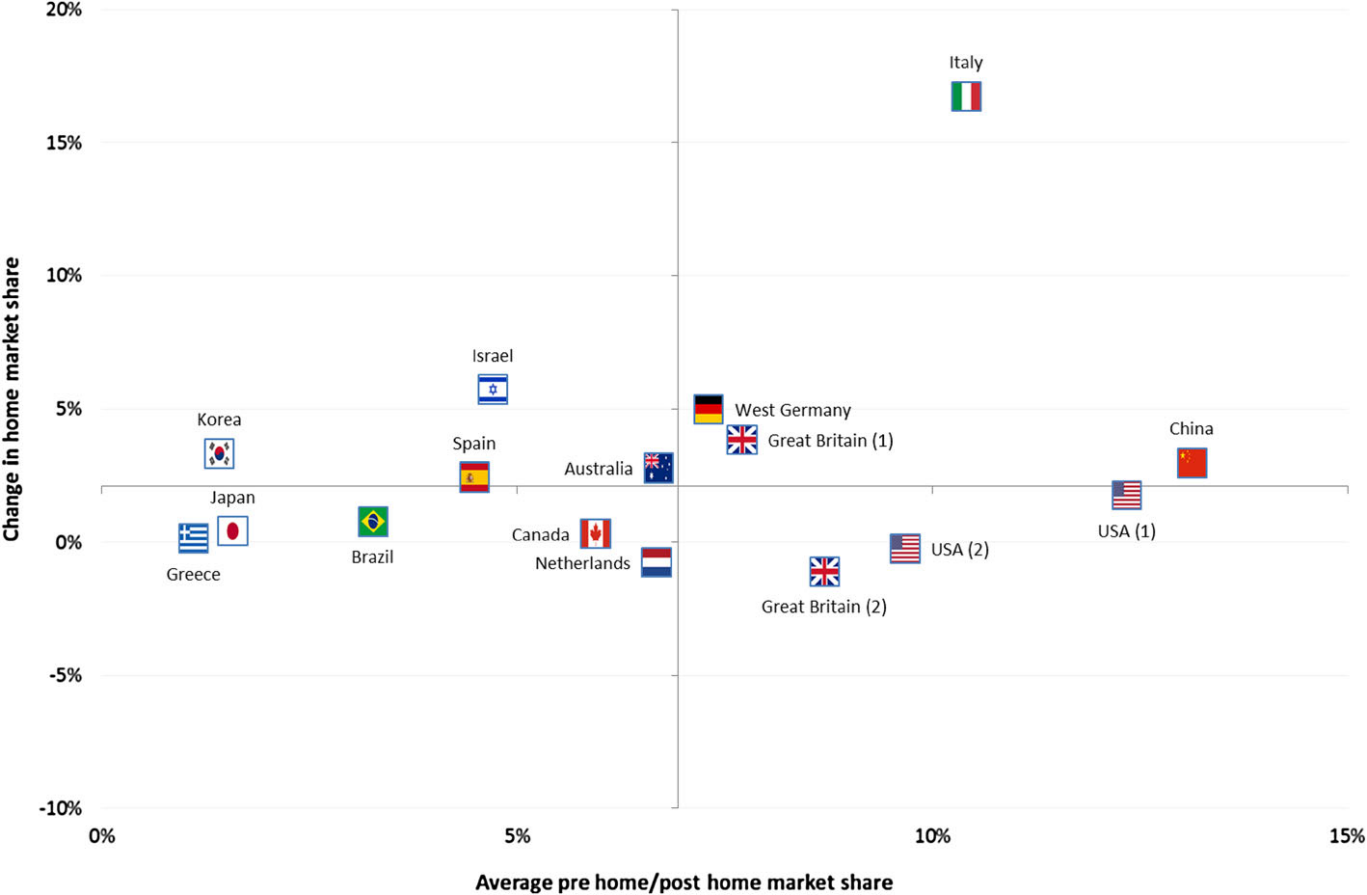
Relationship between away performance and home advantage by nation

Figure 1 plots the average pre-home and post-home market share for each host nation (on the horizontal axis) against their corresponding home advantage scores (on the vertical axis). The axes intersect at the median away performance score across all host nations (6.69%) and the median home advantage score (2.11%). If as suggested by previous research [1, 14] away performance is accepted to be a reliable indicator of overall nation quality at a given point in time, then some mixed patterns emerge. On the one hand, some relatively stronger nations (positioned in the top right quadrant of Fig. 1) exhibit a higher home advantage in comparison with relatively weaker nations (in the bottom left quadrant). Conversely, the magnitude of the home advantage is higher in the case of some relatively weaker nations (in the top left quadrant) relative to some nations with high away market shares (in the bottom right quadrant). Overall, there is no discernable relationship between nation strength and the size of the home advantage effect (*r*_s_ = 0.141, *p* = 0.602).

The prevalence of home advantage was found to vary according to sport. In two sports, athletics and table tennis, a home advantage effect appeared to be present on 13 of the 16 occasions (81.25%). Both archery and wheelchair fencing had a prevalence rate of 50%, whereas the corresponding scores for swimming and wheelchair basketball were 37.5% and 18.8%, respectively. The differences between nations’ home market shares and their corresponding average pre/post-home market shares for each sport are shown in Table 4. Only athletics (*Z* = 2.792, *p* = 0.005), table tennis (*Z* = 3.107, *p* = 0.002), and wheelchair fencing (*Z* = 2.312, *p* = 0.021) returned statistically significant differences between home and away performances.

**Table 4:** Difference between home and away market share of host nations by sport

Year	Host	Athletics (%)	Swimming (%)	Table tennis (%)	Wheelchair fencing (%)	Archery (%)	Wheelchair basketball (%)
1960	Italy	34.13	8.93	24.13	61.90	−2.94	0.00
1964	Japan	−2.86	−0.15	8.64	4.76	5.88	0.00
1968	Israel	10.31	1.61	9.37	7.05	0.00	29.17
1972	West Germany	7.86	−2.23	2.25	5.42	24.65	−8.33
1976	Canada	−0.53	−2.01	3.06	0.00	2.45	0.00
1980	Netherlands	0.46	−4.45	8.29	2.97	−1.73	8.33
1984	Great Britain (1)	7.48	−0.04	2.99	0.00	3.55	0.00
	USA (1)	2.16	−0.20	−2.07	0.00	0.34	−33.33
1988	Korea	2.64	−0.13	8.46	13.10	20.77	0.00
1992	Spain	3.37	1.11	1.18	5.40	3.84	0.00
1996	USA (2)	0.52	−2.00	3.44	0.00	−2.38	4.17
2000	Australia	4.31	1.55	0.00	0.00	0.00	−12.50
2004	Greece	−0.27	0.88	0.00	0.00	0.00	0.00
2008	China	3.75	0.40	12.89	30.97	15.87	0.00
2012	Great Britain (2)	0.93	−1.87	0.86	−1.19	−12.04	−8.33
2016	Brazil	2.01	−0.34	2.87	−4.17	0.00	0.00

**Fig. 2:**
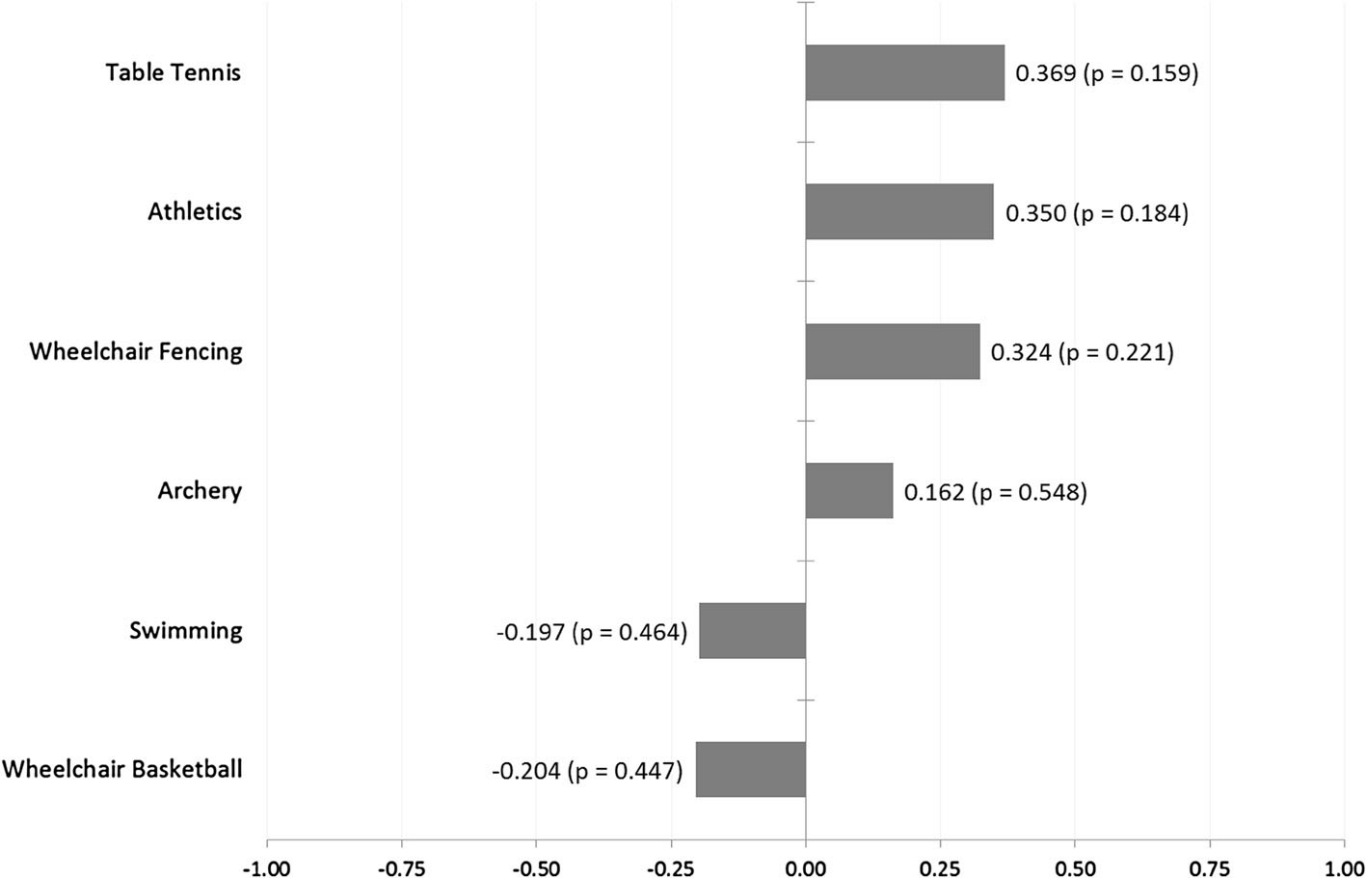
Correlation between average pre/post-home market share and home advantage by sport

The Spearman rank correlation coefficient for each sport showing the nature of the relationship between the size of the home advantage in a given sport and the strength (away performance) of nations in that sport is shown in Fig. 2. None of these correlations were found to be statistically significant at the conventional levels.

## Discussion

### Positioning the research and its findings

#### Research context

Previous home advantage research [4, 5] suggests that ‘performance outcomes’ in sport (primary, secondary, and tertiary) are a function of ‘game location’ (i.e., home or away), four ‘game location factors’ that differentially impact on teams competing at home or away from home (crowd, learning/familiarity, travel, and rules), and the ‘critical psychological, physiological and behavioural states’ of competitors and coaches. Given the paucity of research on home advantage in para-sport events, our study was concerned first and foremost with determining the prevalence and size of the home advantage effect in context of the Summer Paralympic Games using recognised techniques. In other words, we investigated the impact of ‘game location’ on ‘primary performance outcomes’ in the competition. A standardised measure of performance—market share—was utilised to compare the level of medal success achieved by nations when competing at home and away from home.

#### Country-level findings

Our results show that host nations in the Summer Paralympic Games generally performed better at home than away from home and that the difference between home and away performances was statistically significant (*p* < 0.01). In other words, strong evidence of a home advantage effect at overall country level was identified. This finding is consistent with what is known about home advantage in the Winter Paralympic Games [1]. The confirmation of an overall home advantage effect in our study is also in line with research relating to other international multi-sport competitions at which the home advantage phenomenon has been investigated, including the Summer Olympic Games, the Winter Olympic Games as well as the Commonwealth Games [7, 8, 10, 14–16].

#### Sport-specific effects

Our results also point to sport-specific variations in home advantage in the Summer Paralympic Games. Across the six sports to be held in every edition of the competition to date, only athletics, table tennis, and wheelchair fencing exhibited a significant home advantage effect (*p* < 0.05). Evidence of home advantage in archery, swimming, and wheelchair basketball was either weak or inconclusive. Variations in the prevalence and size of the home advantage effect between different sports have also been observed in other studies of multi-sport events [1, 8, 9, 15, 16]. For instance, Wilson and Ramchandani [1] found evidence of home advantage in the Winter Paralympic Games in the sports of alpine skiing and cross country skiing but not in biathlon, curling, ice sledge hockey, and ice sledge skating.

#### Relationship with nation strength

We found that the size of the home advantage effect was not significantly correlated with the quality or strength of the host nation. This finding indicates that stronger nations in the Summer Paralympic Games did not benefit from a larger home advantage effect. One the one hand, our findings are in stark contrast to Wilson and Ramchandani’s analysis of the Winter Paralympic Games [1], according to which home advantage is typically larger in the case of stronger nations. Conversely, the apparent lack of a systematic relationship between nation strength and home advantage in our study is in line with previous research set in the context of another multi-sport event, the Commonwealth Games [14].

### Consideration of causal factors

While our results confirm that home advantage is prevalent in the Summer Paralympic Games at an overall country level and within specific sports, they do not explain why such an effect might occur. As a first step in this direction, we consider below potential influences based on evidence drawn from academic literature.

#### Physiological influences

A potential physiological source of home advantage is related to travel across time zones for competitors from non-host nations [17], which can lead to jet lag and affect athletic performance [18]. Moreover, the severity of jet lag and subsequent recovery is a function of the number of time zones crossed [19]. In fact, the problems of jet lag can last for over a week if the flight crosses 10 time zones or more, and they can reduce performance and the motivation to train effectively [20]. Research by Ramchandani and Wilson [14] found that the performance of nations in the Commonwealth Games was negatively correlated with the number of time zones that they had to traverse. However, in a previous study of the Winter Olympic Games, the number of time zones and direction of travel produced no discernible trends or differences in performance [8]. Another physiological factor that may contribute to home advantage according to previous research is elevated testosterone levels of host nation competitors [21].

#### Psychological influences

Home advantage in some international multi-sport events has been documented as a result of referee bias in sports that require subjective scoring or judgments [8–10, 12, 15, 16]. In their analysis of Great Britain’s performance in the Summer Olympic Games in London 2012, Nevill et al. [10] reported that crowds appear to have had an important effect on influencing officials to favour the home athletes and hence increase their medal winning capacity. However, in our study, athletics and table tennis are objectively judged sports, and therefore, referee bias engineered by home crowds is unlikely to be the source of home advantage in these Summer Paralympic sports.

Wheelchair fencing is a combat sport and as such may require some subjective input from judges, which might explain some of the observed home advantage effect in our study. The prevalence of home advantage in combat sports (including fencing) has previously been documented during the Olympic Games [12]. Previous research also indicates that the home crowd pressure can influence refereeing decisions in team sports [22]. However, the prevalence of home advantage in wheelchair basketball in our study was very low and not statistically significant (*p* > 0.05).

#### Learning factors

Home competitors’ familiarity with local conditions or the venue is a game location factor that is sometimes associated with home advantage. For example, Bray and Carron [23] acknowledged that the beneficial effects of familiarity with the venue could contribute to the home advantage in alpine skiing. This observation was supported by Balmer et al. [8] who noted that the effect of familiarity with local conditions in the Winter Olympic Games was most evident in alpine skiing, where the potential for variation is at its greatest. However, the three sports in which home advantage was observed in our study appear to have little (if any) potential for variation in local conditions in contrast to sports such as alpine skiing. Hence, the relative effect of learning factors on home advantage in this case is likely to be negligible, if not non-existent.

#### Increased funding

From a strategic point of view, there is evidence that countries increase their level of investment in elite sport prior to hosting the Olympic and Paralympic Games [13]. Indeed, in the 4 years leading up to the Beijing 2008 Games, UK Sport (the agency in charge of maximizing the performance of athletes representing Great Britain in the Olympic and Paralympic Games) spent £29.54 million on Summer Paralympic sports alone and this figure increased by nearly 67% to £49.25 million in the 4 years leading up to the London 2012 Games, when Great Britain was the host nation (http://www.uksport.gov.uk/our-work/investing-in-sport/historical-funding-figures).

Despite this considerable growth in elite sport funding, there was only a marginal improvement in Great Britain’s market share in the Summer Paralympic Games between its pre-home edition in 2008 (7.55%) and its home edition in 2012 (7.62%). Nevertheless, it is still conceivable that increased financial support can contribute to home advantage, particularly when considering that Great Britain’s market shares in the sports of athletics and table tennis at its home edition in 2012 improved by around three percentage points each in comparison with 2008.

## Conclusion and future work

Building on a recent study [1], this research has extended the evidence base of home advantage in the context of para-sport events. In summary, there is clear evidence of a home advantage effect in the Summer Paralympic Games at country level and for certain sports. Although we discuss some possible reasons for these findings, the causes of home advantage in the competition remain unclear. This is both a limitation of the current study and a direction for future research. Once the underlying factors affecting home advantage in the competition are better understood, relevant practical applications can be proposed. Beyond the empirical investigation of the factors that contribute to our results, future research should utilise similar methods to compare and contrast the results from this study with the Summer Olympic Games. A comparative analysis of this type would provide further insight into any differences in home advantage between para-sport events targeted at athletes with a disability and events that feature able-bodied competitors.
